# Royal Mineral Water Hospital, Bath

**Published:** 1916-02-19

**Authors:** 


### SOME HOSPITAL CHAPELS.

### ROYAL MINERAL WATER HOSPITAL, BATH.

ALTHOUGH the Royal Mineral Water Hospital at Bath was founded in 1737, for many years the institution was not provided with a chapel, and the services were performed in the wards; in 1860, however, an additional wing was added to the hospital, and of this the chapel formed an important part. There is an ante-chapel, separated from the chapel itself by a screen of three arches; the roof is vaulted and supported by composite pilasters, the capitals of which are enriched by carving in stone. The organ stands on the north side, and facing it is a stained-glass window which has for its subject the Good Samaritan; this was erected by a number of the governors at their own expense in memory of James Snaith Brymer of Bath, who was a generous supporter of the hospital, and a donor of £500 to be specially applied to the decoration of the chapel. The chapel measures some 55 ft. by 25, and has on the south side five two-light windows filled with stained glass; columns of Devonshire marble adorn each window, and their caps are enriched by devices carved in stone. The ceiling is of lacunary work after the old church of Constantine at Jerusalem; its four transverse ribs spring from corbels, carved with heads of the Apostles St. Peter, St. John, and St. Stephen, King David, Moses, and three archangels, and the panels of the ribs are relieved by carvings of lilies and olive branches, while around the walls and underneath the cornice appear seven sentences from the "Te Deum."

The apse, which is approached by two steps, is lighted by seven stained-glass windows, the subjects of which were selected for their references to water: they are—the Baptism of Christ in the River Jordan, our Saviour at the Pool of Siloam, our Lord washing the feet of His Disciples, the Baptism of the Eunuch by St. Philip, Christ and the woman of Samaria, Naaman the Syrian in the waters of the Jordan, and Moses striking the rock. The caps of the marble columns of these windows exhibit the emblems of the Passion, and we see portrayed the chalice, the crown of thorns, the cross with the Passion flower, wheat and grapes, the spear, hyssop, and scourge, the hammer and pincers, and the nails.

The half-dome of the apse is coloured blue, and the arch is supported by four columns of marble, on the caps of which are the symbols of the four Evangelists.

The pulpit is of white lias from Clandown; the corbel supporting it is formed of two storks surrounded by bulrushes.

Accommodation for some 150 persons is provided in the chapel, which, it may be mentioned, was designed by John Elkington Gill, of Bath, and dedicated by the Bishop of Bath and Wells on July 11, 1861. It is a worthy example of happy design and perfect execution, and forms a most fitting and enduring memorial of James Snaith Brymer.

The institutional world can boast of many records of long service, and in that connection it is more than probable that the honour in length of service as a hospital chaplain belongs to the Rev. Thomas Tyers, who for forty-three years has ministered to the patients of the Royal Mineral Water Hospital, Bath. Mr. Tyers, who was educated at Caius College, Cambridge, and is a Master of Arts, was ordained in 1869 to the curacy of Widcombe, Bath, where he remained until 1872, in which year he was appointed to his present position.

